# Caloric restriction induced epigenetic effects on aging

**DOI:** 10.3389/fcell.2022.1079920

**Published:** 2023-01-13

**Authors:** Jingfang Zhai, William H. Kongsberg, Yinbo Pan, Canhua Hao, Xiaojing Wang, Jie Sun

**Affiliations:** School of Pharmacy and Pharmaceutical Sciences & Institute of Materia Medica, Shandong First Medical University & Shandong Academy of Medical Sciences, NHC Key Laboratory of biotechnology drugs (Shandong Academy of Medical Sciences), Key Lab for Rare & Uncommon Diseases of Shandong Province, Ji'nan, Shandong, China

**Keywords:** caloric restriction, aging, epigenetic, autophagy, drugs

## Abstract

Aging is the subject of many studies, facilitating the discovery of many interventions. Epigenetic influences numerous life processes by regulating gene expression and also plays a crucial role in aging regulation. Increasing data suggests that dietary changes can alter epigenetic marks associated with aging. Caloric restriction (CR)is considered an intervention to regulate aging and prolong life span. At present, CR has made some progress by regulating signaling pathways associated with aging as well as the mechanism of action of intercellular signaling molecules against aging. In this review, we will focus on autophagy and epigenetic modifications to elaborate the molecular mechanisms by which CR delays aging by triggering autophagy, epigenetic modifications, and the interaction between the two in caloric restriction. In order to provide new ideas for the study of the mechanism of aging and delaying aging.

## 1 Introduction

**FIGURE 1:**
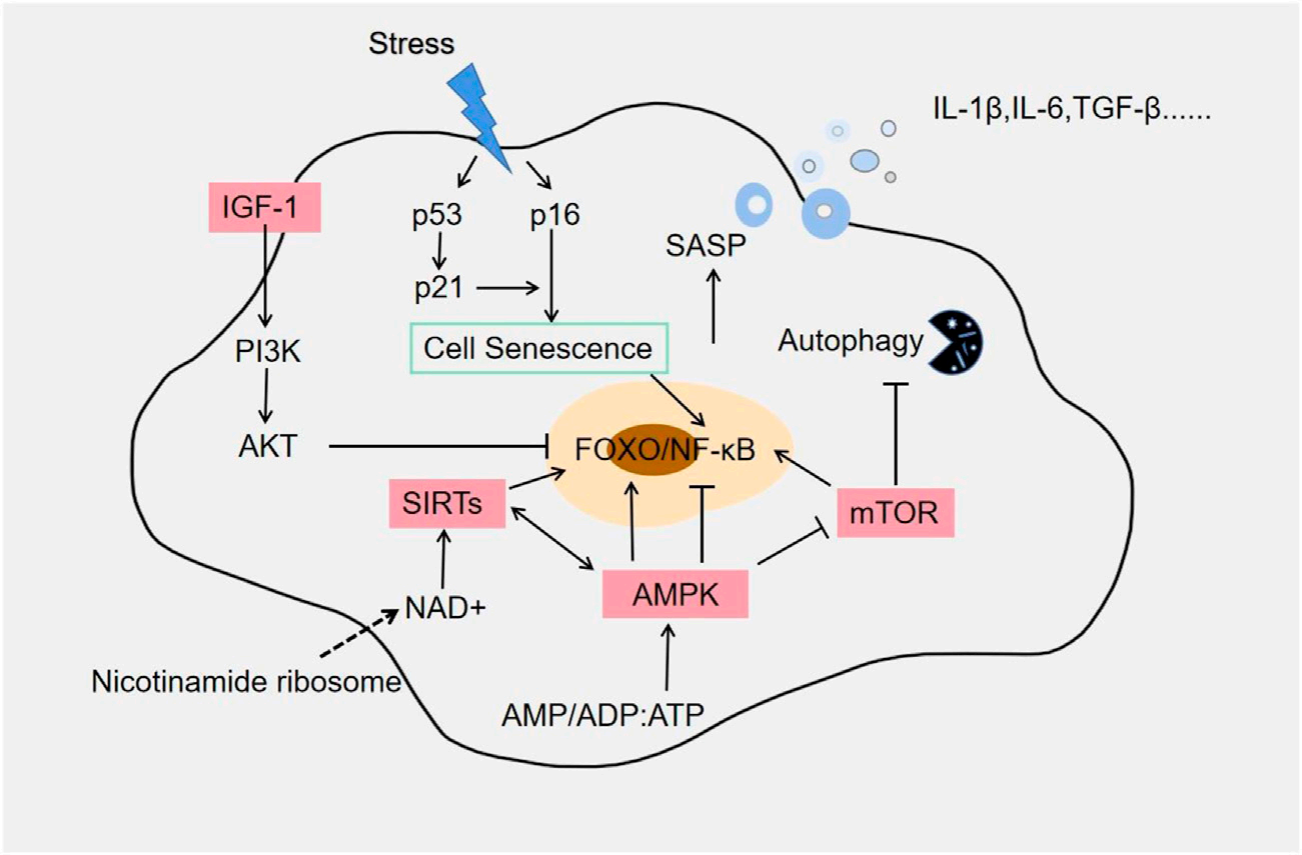
Aging-related signaling pathways. Most pathways establishing the SASP phenotype appear to converge on activation of the NF-κB and FOXO pathways. Rapamycin (mTOR) pathway is also an important node in SASP regulation, and it is a key role in the aging process. Elimination of senescent cells reduces many pro-inflammatory factors, such as IL-6, IL-1α, and TNF-α. Autophagy is a major lysosomal degradation pathway, and CR activates autophagy and may act as an anti-aging agent by removing damaged proteins and organelles such as mitochondria.

With the continuous improvement of living and medical care, the average life expectancy of mankind continues to grow. Population ageing has become a major public burden in the 21st century. It is predicted that the global elderly population over the age of 60 may exceed 2 billion by 2050 (Wyss-Coray, 2016). Aging is an activity that accompanies life development and is caused by the accumulation of many different insults, including oxidative stress, telomere loss, epigenetic changes, metabolic dysfunction, loss of certain tumor suppressor genes or excessive activation of oncogenes, and mitochondrial dysfunction (McHugh and Gil, 2018). These cumulative injuries gradually impair biological function, reduce organism resistance and fitness, gradually decline organ function over time, and ultimately lead to organism death. Studies have shown that various external environments can cause cellular senescence. Cellular senescence is an irreversible state of growth arrest characterized by hypertrophy and secretion of various bioactive molecules, a phenomenon defined as the Senescence Associated Secretory Phenotype (SASP). SASP is one of the key markers of aging (Figure 1). Excessive accumulation and decreased regenerative capacity of senescent cells are associated with chronic inflammation. Aging is not only accompanied by a decline in health status, but is further associated with an increase in disease incidence and becomes an important risk factor. Therefore, in order to reduce the accumulation of senescent cells and delay the aging process, it is still urgent to discover novel anti-aging methods.

Calorie restriction (CR) is currently the most effective non-genetic intervention to delay the aging phenotype, and different studies have shown that CR reduces aging markers in mouse organs and human colonic mucosa (Ogrodnik et al., 2017; Fontana et al., 2018). CR, also known as dietary restriction, usually refers to a daily reduction of 20%–50% of the total calories of the diet when providing the organism with adequate nutrients (e.g., essential amino acids, vitamins, *etc.*) to ensure that the organism does not develop malnutrition (Colman et al., 2009). As a simple and reproducible life-prolonging dietary manipulation, it has been widely used in aging research. Observational studies have shown that CR also has beneficial effects on human longevity. Among many well-known anti-aging strategies, CR has been identified as one of the effective interventions to combat the aging process and age-related pathological diseases (e.g., diabetes, kidney disease, cardiovascular disease, cancer, Alzheimer‘s disease, *etc.*) (Dan Dong et al., 2017; Mattison et al., 2017; Pulakat and Chen, 2020). Thus, CR may influence the aging process by favorably affecting human health, and it is of considerable importance to be used to identify the underlying signaling mechanisms of aging. Mechanisms that extend lifespan through CR modulation of autophagic function or epigenetic modifications have also gradually been explored and focused. In this paper, we focus on autophagy and epigenetics to explore the molecular mechanism of caloric restriction delaying aging and its interactive relationship, providing a basis for the study of aging.

## 2 CR promotes autophagy and delays aging

Because cell damage is one of the main triggers of aging, it is highly likely that CR reduces the production of senescent cells by repairing/eliminating already existing damage, for example, increasing autophagy (Il’yasova et al., 2018; Yang et al., 2016). Autophagy is a process in which cells degrade their own components and can circulate and utilize them, which plays a key role in delaying aging. In addition, the development and progression of several human diseases are strongly associated with age-related defects in autophagy (Saxton and Sabatini, 2017). Age-induced autophagy disorders further contribute to the development of disease. Levels of autophagy have been shown to decrease with age (Simonsen et al., 2008). Age-related decreased autophagy is associated with various typical aging-related symptoms, including inflammation, cancer, degenerative diseases, *etc.* For example, autophagy can remove larger proteotoxic aggregates that accumulate with age (Tanaka and Matsuda, 2014); reduce the occurrence of age-related neurodegenerative diseases such as Alzheimer’s disease (AD) and Parkinson’s disease (Son et al., 2012); some studies have shown that excessive levels of free fatty acids can hinder the fusion of autophagosomes with lysosomes and thus inhibit autophagy, suggesting that autophagy is involved in metabolism (Koga et al., 2010). Autophagy may also clear damaged or deleterious cells and reduce the propensity of leukocytes to produce pro-inflammatory cytokines through efficient phagocytosis (Ma et al., 2013; Teplova et al., 2013; Liu et al., 2014). In conclusion, autophagy mechanisms play a critical role in delaying aging, and altered autophagy has emerged as an aging feature in many species.

Through a large number of studies, it has been found that a series of autophagy-related genes (ATG) such as microtubule associated protein light chain 3 (LC3) and p62 encode the corresponding autophagy-related proteins and participate in the whole process of autophagy. Initial identification of ATG was accomplished in *S. cerevisiae*, where autophagy is essential in response to nutrient starvation. Autophagy is regulated by many signaling pathways, such as mTOR (currently considered a key factor), ribosomal protein S6 kinase (S6K), and serine-threonine kinase (Akt). Downregulation of these pathways leads to upregulation of autophagic activity and is a negative autophagic regulatory protein. In contrast, cellular energy status sensing AMPK and deacetylase sirtuin 1 (SIRT1) are positive regulators of autophagy (Revuelta and Matheu, 2017). Whereas cell growth signal insulin-like growth factors −1 (IGF-1), through activation of Akt, leads to activation of TORC1 and inhibits autophagy (Korets et al., 2011).

**FIGURE 2:**
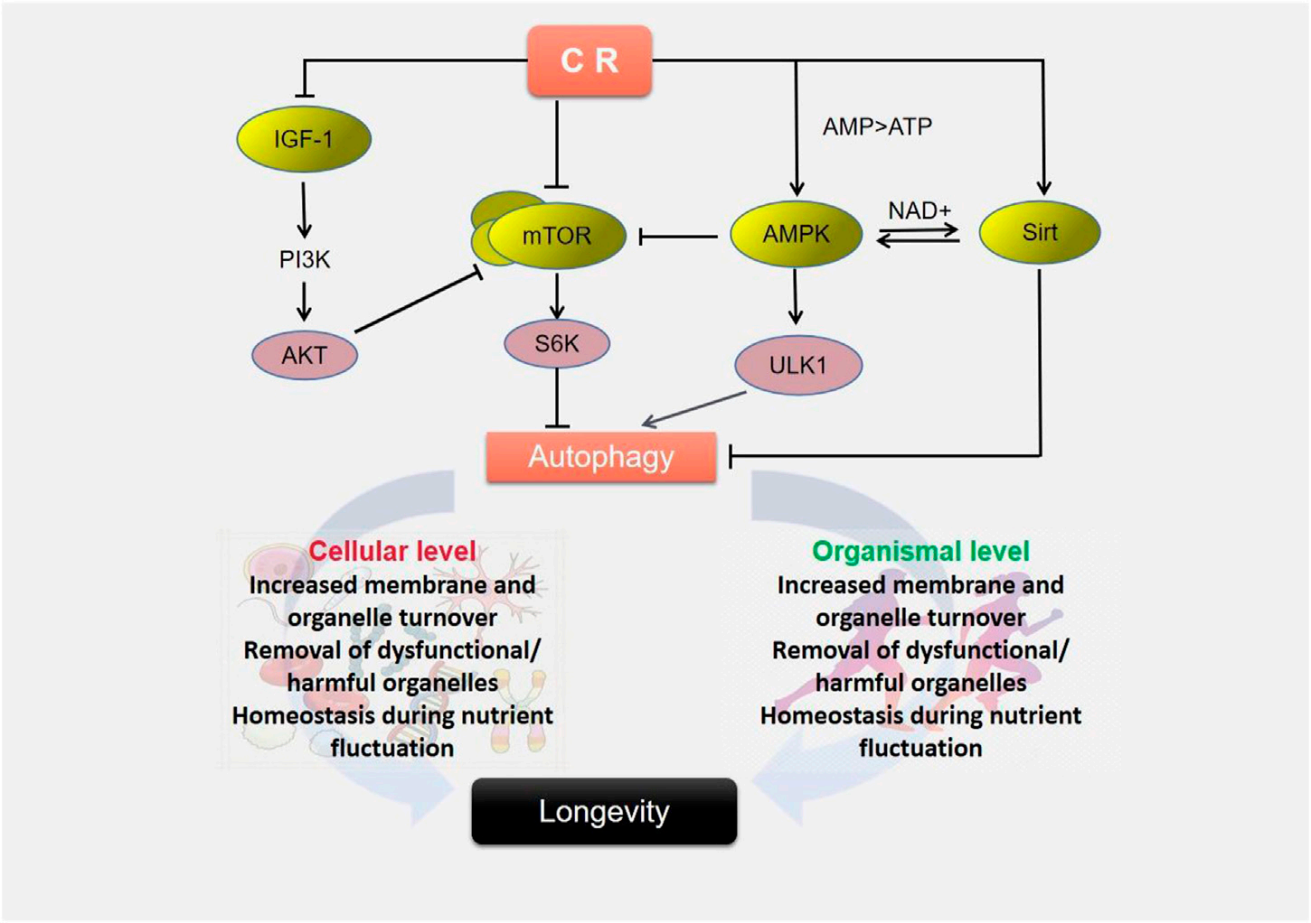
Caloric restriction triggers autophagy to extend lifespan.

CR has already been shown to promote autophagy *in vitro* and *in vivo*, by accelerating the recycling of metabolites and energy *in vivo*, thereby prolonging lifespan (Dutta et al., 2014; Saxton and Sabatini, 2017). Martel et al. suggest that CR can be regarded as mild biological stress in the context of excitatory effects, improving cellular resistance and organ function by activating cellular processes (e.g., autophagy, mitochondrial biogenesis, DNA repair, *etc.*) (Martel et al., 2021). At present, the mechanism of CR promoting autophagy and anti-aging has not been fully studied, but it is mainly divided into two aspects: on the one hand, it achieves the purpose of anti-aging by reducing the levels of glucose and insulin, and on the other hand, it exerts the effect of promoting longevity by regulating multiple signaling pathways in cells, including AMP-activated protein kinase (AMPK), mammalian target of rapamycin (mTOR) and Sirtuins. They form a rather active network of interactions. These signaling pathways not only involve the sensing of cellular energy metabolism status, but also participate in CR-related beneficial effects to varying degrees in different tissues or organs, directly or indirectly regulate autophagy and stress responses, forming a quite active intercrossing network to exert longevity promoting effects (Figure 2).

### 2.1 mTOR

mTOR is a key signaling molecule regulating autophagy and contains two signaling protein complexes, namely mTORC1 and mTORC2. The mTOR pathway is regulated by a variety of cellular signals and plays an important role in lipid and protein synthesis, cell growth and proliferation, induction of autophagy and metabolic regulation (Jung et al., 2010). S6K is a specific molecule downstream of S6 kinase. mTORC1 can block autophagy and regulate protein translation by phosphorylating S6K and 4EBP. When the mTOR signaling pathway is activated, ribosome adhesion to the ER is enhanced, autophagosome membrane formation is inhibited, and autophagy is inhibited. Conversely, inhibition of mTOR signaling pathway activity promotes autophagy (Jung et al., 2010; Saxton and Sabatini, 2017). Thus, mTOR is an important switch that turns off cellular stress resistance and autophagy and turns on protein synthesis. CR can significantly reduce the activity of mTOR and downstream S6K, thereby delaying cellular senescence and prolonging the life span of mice (Schloesser et al., 2015). CR inhibits mTOR signaling and attenuates age-related EMT4 in aging kidneys; it also reduces mTOR and p62 expression and activates autophagy in mice to prevent age-related cognitive dysfunction (Dan Dong et al., 2017).

### 2.2 AMPK signaling pathway

AMPK is one of the central regulators of metabolism in eukaryotic cells and organisms, which can sense the intracellular energy status and maintain the stable operation of cellular physiological activities by sensing changes in intracellular AMP/ATP levels (Hardie et al., 2012). Related studies on model organisms have demonstrated that regulating AMPK signaling can affect the aging process of organisms (Hardie et al., 2012). When CR leads to intracellular undernutrition, ATP synthesis decreases and AMP content increases, allowing AMP/ATP to rise and AMPK to be activated. Activated AMPK regulates new mitochondrial production through peroxisome proliferator-activated receptor gamma coactivator 1 (PGC-1α) transcription, thereby increasing ATP synthesis and increasing ULK1 protein activity, indirectly promoting the development of autophagy. Simultaneously activated AMPK inhibits mTORC1 activity and promotes autophagy. This dual regulation of AMPK plays a key role in many physiological and pathological processes (Abid et al., 2019). Dan et al. found that AMPK/mTOR signaling was downregulated with age in the kidney, and short-term CR and metformin treatment could reverse this phenomenon and prolong the life cycle (Dan Dong et al., 2017). In addition to regulating energy metabolism, AMPK can upregulate the expression of multiple antioxidant enzymes by activating transcription factors such as FOXO3a and Nrf2, improve the antioxidant capacity of cells, and lead to the downregulation of mTORC1 to activate autophagy (Wu et al., 2014). Yang et al. found that *ß*-guanidinopropionic acid (β-GPA) chronically activated AMPK and prolonged *Drosophila* lifespan through the AMPK-Atg1-autophagy signaling pathway (Yang et al., 2015a). Therefore, AMPK, as a hub of intracellular energy control, is also an important triggering mechanism of mTOR pathway and mediates a variety of beneficial biological effects of CR.

### 2.3 Insulin/insulin-like growth factors-1 signaling (IIS)

Relaxation in nutrient perception is an important hallmark of aging. Among them, the IIS signaling pathway is the key to this process and coordinates the growth, differentiation and metabolism of the body in response to changes in environmental conditions and nutrient supply. IGF-1 is an important negative regulator of autophagy. The DAF-2 mutation in *C. elegans* was initially found to result in downregulation of IGF expression and would extend *C. elegans* lifespan approximately twofold compared to common adults. However, autophagy is an essential cellular pathway for *C. elegans* development and lifespan extension. If autophagy is inactivated, this life-prolonging manipulation usually fails (Kenyon et al., 1993). Under normal physiological conditions, IGF-1 binds to its specific receptor and activates phosphatidylinositol-3-kinase (PI3K) to Akt. Activated Akt phosphorylates several cellular proteins, including (forkhead box O, FOXO), mTOR, and activated B-cell nuclear factor κB (NF-κB) (Manning and Cantley, 2007). The experiment showed that serum IGF-1 in mice decreased with age after 9 months (Hadem and Sharma, 2016); persistent low levels of IGF-I in mouse brains contributed to longer lifespan and reduced age-related mortality (Kappeler et al., 2008). CR has also been shown to reduce circulating levels of IGF-1, insulin, or glucose in humans. In addition, decreased circulating levels of IGF-1 can inhibit PI3K/Akt signaling, improve cancer resistance, and enhance DNA repair capacity (Lin et al., 2017; Piscitello et al., 2018). Studies from nematodes to humans have shown that the insulin signaling pathway is evolutionarily highly conserved and plays an important role in regulating aging in organisms, and insulin/IGF-1 (Bartke, 2008), a signaling pathway that regulates growth and development in organisms, is a potential target for anti-aging (Kapahi et al., 2004).

### 2.4 Sirtuin

The Sirtuin family is a histone deacetylase that catalyzes deacetylation of various proteins including histones in a NAD^+^ dependent manner. Seven sirtuin orthologues have been identified in mammals, namely SIRT1 to SIRT7. Mammalian sirtuin can interact with proteins such as p53, PGC-1α, NF-κB, and FOXO to regulate cellular stress, metabolism, and apoptosis (Wang et al., 2020). SIRT1 and its homologs (silent information regulator 2, SIRT2) are longevity factors that delay aging and prolong lifespan. In starvation or low-energy states, SIRT1 activation is regulated by AMPK, which increases lifespan by increasing NAD^+^ levels and activating SIRT1 (Zullo et al., 2018). At the same time, SIRT1 can also activate the AMPK pathway and inhibit mTOR by promoting deacetylation of hepatic kinase B1 (LKB1) upstream of AMPK (Lan et al., 2008). In addition, SIRT1 can promote autophagy by activating Atg5, Atg7, Atg8, and LC3 (Lopez-Lluch and Navas, 2016; Revuelta and Matheu, 2017; Saxton and Sabatini, 2017). Thus, SIRT1 regulates autophagy. CR was found to increase SIRT1 levels in the brains of Machado-Joseph disease (MJD) mice, significantly improve neuropathology, reduce neuroinflammation and activate autophagy (Cunha-Santos et al., 2016). This means that SIRT1 plays a critical role in controlling CR-mediated changes in gene expression and cellular function, thereby improving health and longevity.

The role of SIRT2 in CR-mediated lifespan extension depends on cAMP-PKA and casein kinase 2 (CK2) signaling in yeast (Kang et al., 2015). In mice treated with CR for 18 months, SIRT2 expression levels were significantly increased in their white adipose tissue and kidneys (Wang et al., 2007). In addition, SIRT2 is able to prevent chromosomal instability during mitosis, is involved in a variety of metabolic processes, and also plays an important role in delaying aging and aging-related diseases. The role of SIRT2 in tumorigenesis is complex. Animals genetically deficient in SIRT2 have been reported to exhibit genomic instability and chromosomal abnormalities and are prone to tumor development (Kim et al., 2011; Serrano et al., 2013). However, SIRT2 regulates deacetylation and activation of AKT1, which in turn affects glycogen synthase kinase-3b/b-catenin (GSK-3β/β-catenin) signaling pathway and promotes epithelial to mesenchymal transition (Chen et al., 2013). Thus, inhibition of SIRT2 may have broad anticancer effects in a variety of human cancer cells and mouse breast cancer models (Jing et al., 2016).

In addition to SIRT1 and SIRT2, SIRT3 is also a longevity factor and plays an important role in counteracting oxidative stress and preventing cellular senescence and tumorigenesis (Alhazzazi et al., 2011; Jing et al., 2011; Haigis et al., 2012; Albani et al., 2014). Li et al. showed that SIRT3 expression levels in the myocardium of aged mice were significantly lower than those of young mice (Li et al., 2018a). Increasing evidence suggests that decreased SIRT3 expression is associated with multiple age-related diseases. It has been shown that SIRT3 deficiency is associated with cardiac aging, at least in part because it inhibits mitochondrial phagocytosis and impacts mitochondrial function associated with oxidative stress and energy metabolism (Li et al., 2018a). However, the mechanism of SIRT3 in regulating cardiac aging and mitochondrial function remains to be further investigated. Similarly, SIRT3 deletion also accelerated ovarian aging, with increased expression of aging and inflammation-related genes (p16, p21, IL-1α, and IL-1β) (Zhu et al., 2022). SIRT3 also inhibits tumor growth by deacetylating glutamic-oxaloacetic transaminase 2 (GOT2). Acetylation of GOT2 stimulates NADPH production and promotes pancreatic cell proliferation and tumor growth *in vivo* (Yang et al., 2015b). In addition, sirtuins play an important role in reducing oxidative damage (Someya et al., 2010). SIRT3 increases the expression of manganese superoxide dismutase (MnSOD) and catalase (CAT) by deacetylating the transcription factor FOXO3a, and finally decreases ROS levels in the myocardium (Aldakkak et al., 2008; Sundaresan et al., 2009). PGC-1α and FOXO3a are two transcription factors that synergistically regulate antioxidant gene expression. SIRT4, SIRT6 also have tumor suppressive effects (Sebastian et al., 2012; Jeong et al., 2013; Yang et al., 2015b). SIRT6 overexpression could reduce the activity of inflammation-related transcription factor NF-κB, thereby inhibiting cellular senescence; and cellular senescence was accelerated when SIRT6 was knocked down (Zhang et al., 2016). CR can activate SIRT3 and SIRT6. Mechanistically, PGC-1α activates SIRT3 transcription by coactivating estrogen-related receptor alpha (ERRα), whereas CR increases SIRT1 protein and NAD^+^ levels and then activates PGC-1α. SIRT1 forms a complex with FOXO3a and NRF1 on the SIRT6 promoter and positively regulates SIRT6 expression (Kim et al., 2010). Thus, CR triggers an increase in SIRT1 activity, which induces expression of SIRT3 and SIRT6. In summary, further studies are needed to investigate the effects of different sirtuin proteins in CR mediation and to elucidate their underlying molecular mechanisms.

## 3 CR delays aging through epigenetic modifications

**FIGURE 3:**
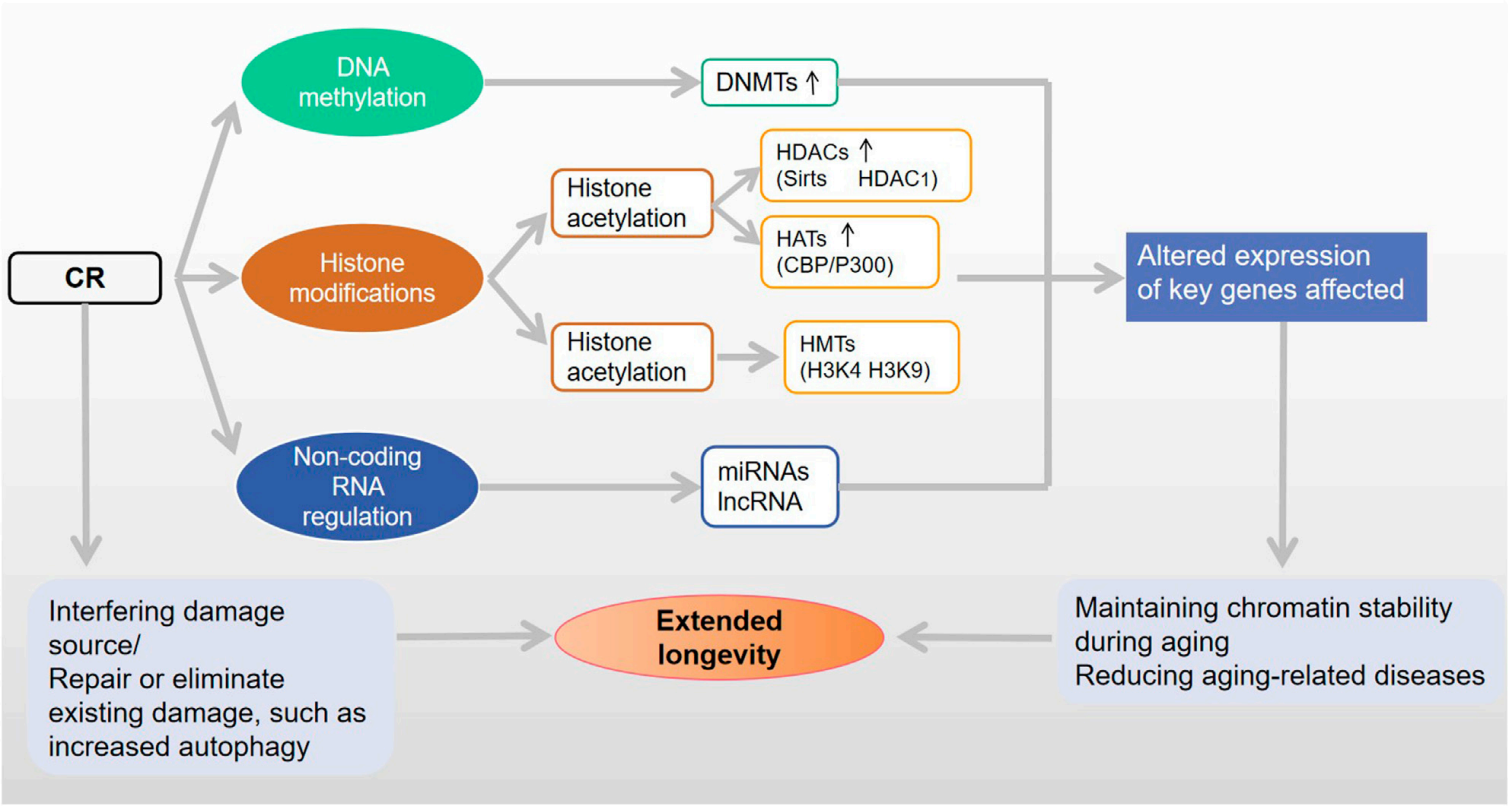
CR pathways to extend lifespan through epigenetic modifications.

Epigenetics refers to heritable changes in gene function in the absence of changes in DNA sequence. Aging is a multifactorial process determined by environmental factors, and Kumar points out that epigenetic changes are also one of the important factors leading to aging (Kumar and Lombard, 2016). Genotypes play an important role in aging and have a significant impact on longevity as well as aging-related diseases. It has been suggested that there may be an association between epigenetics, CR and biological longevity (Hanneke and van Leeuwen, 2012). It has been experimentally shown that epigenetically predicted age is significantly lower in animals under CR conditions (Maegawa et al., 2017; Petkovich et al., 2017; Wang et al., 2017; Thompson et al., 2018). Three different types of epigenetic modifications: DNA methylation, histone modification, and non-coding RNAs (ncRNAs) regulation. Currently there are many studies on DNA methylation and histone modification (Figure 3). These mechanisms play critical regulatory roles in many physiological processes and become important targets for studying delaying aging, improving, and preventing age-related diseases.

### 3.1 DNA methylation

DNA methylation is one of the major epigenetic modifications. DNA methylation refers to covalent bonding of a methyl group at the carbon 5 position of cytosine at genomic CpG dinucleotides under the action of DNA methyltransferases (DNMTs). DNA methylation patterns are dynamically regulated by at least three independent DNMTs: DNMT1, DNMT3a, and DNMT3b (Okano et al., 1999; Goll and Bestor, 2005). DNA methylation patterns are dynamically regulated by at least three independent DNMTs: DNMT1, DNMT3a, and DNMT3b. DNMT1 plays a maintenance function during cell division and maintains the stability of DNA methylation status, while DNMT3a and Dnmt3b are mainly involved in the process of re-methylation (Okano et al., 1999; Goll and Bestor, 2005). Numerous studies have shown that genomic DNA methylation patterns are significantly remodeled during aging, and DNA methylation levels at specific CpG sites can predict the aging status of cells (Brunet et al., 1999), and it can be said that methylation levels at DNA-specific sites are biological markers that can reflect aging (Dozmorov et al., 2017).

As early as the 1990s, DNA methylation was experimentally shown to be one of the molecular markers of aging affected by CR (Chen et al., 2017). A series of studies have pointed out that CR plays a protective role against age-related DNA methylation changes in different tissue types (Lardenoije et al., 2015; Kim et al., 2016; Hahn et al., 2017; Hadad et al., 2018; Sziraki et al., 2018). CR can increase the activity of DNMT1, prevent the downward trend of methylation, and delay aging (Maddock et al., 2020). Leonidas et al. showed that the aging process in mice is associated with epigenetic changes, and that the beneficial effects of CR may be mediated through methylation and hydroxymethylation of DNA (Leonidas et al., 2012). Gensous et al. showed that CR is involved in the regulation of methylation at age-related ribosomal RNA (rRNA) loci, thereby providing an epigenetic readout of CR‘s pro-longevity effect (Gensous et al., 2020). Expression of rRNA is one of the major determinants of translation rate, and epigenetic modifications contribute to its regulation. Remodeling of DNA methylation patterns associated with CR may play a critical role in aging-related diseases. For example, the expression level of neurotensin 1 (Nts1) in mice fed CR diet was significantly increased within 1 month, while the gene expression of CpG sites in the promoter region of Nts1 gene was significantly decreased in mice fed normal diet, which indicated that CR had a long-term effect on the memory of cells (Unnikrishnan et al., 2017). CR also altered DNMT3a levels in the hippocampus of mice and improved brain function in mice during aging (Chouliaras et al., 2011). In the kidneys of aged rats, CR was able to attenuate age-related methylation changes in the promoters of genes associated with inflammation, diabetes, *etc.* (Kim et al., 2016). CR also reduced DNA hypermethylation at the E2F-1 (active transcription factor for p16INK4a) binding site in nurse cells, thereby preventing downregulation of p16INK4a (Tramontano et al., 2020). The p16INK4a gene is associated with cellular senescence regulation and accumulates during aging (Ressler et al., 2006; Li and Tollefsbol, 2011). In addition, a study in obese subjects showed that short-term CR intervention resulted in DNA methylation changes in genes such as ATP10A, WT1, and TNF-a and reduced lipid metabolism gene expression, thereby delaying aging (Hahn et al., 2017) (Table 1). These genes may serve as early indicators of metabolic effector responses. These lines of evidence suggest that CR helps prevent age-related diseases and induces increased longevity.

**TABLE 1:** Effect of caloric restriction on age-related epigenetic modifications.

Model	Biological tissue	Impact	References
Mice	Whole Tissue	Nts1 showed significantly higher expression levels within 1 month	Unnikrishnan et al. (2017)
Mice	Hippocampus	Regulating DNMT3a levels, preventing age-related increases in HDAC2, and improving brain function in mice during aging	Chouliaras et al., 2011; Chouliaras et al., 2013
Female mice	Liver	Attenuates age-related methylation changes in gene promoters associated with inflammation, diabetes*etc.*	Hahn et al. (2017)
Obese humans	_	Short-term CR intervention leads to DNA methylation changes in genes such as ATP10A, WT1, and TNF-a, reduces lipid metabolism gene expression, and delays aging	Hahn et al. (2017)
SAMP8 Mice	cerebrum	As H3K27me3 increases, aging phenomena accelerate and premature neurodegeneration develops	McCauley and Dang, (2014)
Normal diploid WI-38, MRC-5 and IMR-90 human fetal lung fibroblasts	_	Glucose restriction directly activates SIRT1, leading to chromatin remodeling of the p16INK4a gene promoter and decreased expression of this gene, and significantly prolonged cell life span	Pazin et al. (2011)
*C. elegans*	_	Increased H3K27 demethylase UTX-1 activity, increased expression of IIS signaling pathway genes and loss of H3K27me3	Jin et al. (2011)
Aged Mice	_		Acetylation of histone H3 Lys9 increased	Cao et al. (2018)
yeast	_	Nαt4 expression is significantly reduced, histone H4 N-terminal acetylation is lost, and yeast lifespan is extended	Molina-Serrano et al. (2016)
Mice	Serum	Elevated levels of numerous miRNAs	Dhahbi et al. (2013)
Mice	cerebrum	Increases in miR-181a-1*, miR-30e, and miR-34a were counterbalanced, resulting in increased expression of Bcl-2 and decreased apoptosis	Ayyadevara et al. (2013)
Aged Rats	Cerebral cortex	Increased miR-98–3p expression	Wood et al. (2015)

### 3.2 Histone modification

Histone modification is an important field of epigenetic research. Histone is a general term for basic proteins present in the chromatin of eukaryotic cells and prokaryotic cells, which bind to DNA and form nucleosome structures together with DNA. Histone modifications include acetylation, phosphorylation, methylation and ubiquitination of histones. Each modification, either alone or in combination with other modifications, regulates cellular aging and longevity. Among known histone modifications that influence longevity processes, acetylation and methylation are most prominent.

#### 3.2.1 Methylation of histones

Methylation of histones is catalyzed by histone methyltransferases (HMTs) that bind three methyl groups to arginine or lysine residues at the N-terminus of histones. Depending on the binding site, gene expression can be affected differently, that is, promoting or inhibiting gene transcription. It is generally believed that methylation modifications of H3K4, H3K36, and H3K79 promote the expression of genes, while methylation modifications of H3K9, H3K27, and H4K20 inhibit the expression of related genes (Wu et al., 2007; Sadakierska-Chudy and Filip, 2015). Mono-, di-, or trimethylation of lysine residues also differentially regulates genes. Methylation of proteins tends to be suppressed with increasing age. Decreased H3K4 methyltransferase activity was found in astrocytes from older women (Chisholm et al., 2015). In normal cells, methylation modification sites H3K9c and H3K27 of histones are also inversely proportional to age (Pal and Tyler, 2016). In brain tissue from SAMP8 mice, mice developed accelerated aging and premature neurodegeneration as H3K27me3 increased (McCauley and Dang, 2014) (Table 1). Increased H3K9me3 may therefore hinder remodeling of heterochromatin, leading to persistent DNA damage, which in turn leads to early aging and shortened lifespan; while H3K36me3 has been found to promote longevity (Pal and Tyler, 2016). H4K20me3 has been reported to increase with age (Biron et al., 2004). In cancer cells, loss of expression of the methyltransferase Suv4-20h2, which is relatively specific for H4K20 methylation, contributes to reducing H4K20me3 (Tsang et al., 2010).

CR may also modulate aging through histone methylation modifications. Under CR conditions, the H3K4 locus is trimethylated, chromosome conformation is changed, hTERT is activated and expressed, and cell life span is prolonged (Li and Tollefsbol, 2011). Expression of p16 is silenced by trimethylation modification at H3K27. The experiments by Li et al. also demonstrated that the decreased expression of p16 in cells under glucose inhibition was partly due to the induction of chromatin remodeling through effects on their promoter methylation, thereby extending cell lifespan (Li and Tollefsbol, 2011). During aging in *C. elegans*, H3K27 demethylase UTX-1 activity increases, which in turn causes an increase in gene expression and loss of H3K27me3 in the IIS signaling pathway (Jin et al., 2011) (Table 1). Different methylation modifications of histones by CR, combined with different transcription factors, co-regulate aging-related protein expression, thereby delaying aging. Currently, CR has been less studied to regulate aging through methylation modification of histones and is generally associated with DNA methylation. Pal et al. proposed that methylation of HTM-directed H3K9 sites must have DNMT3b-dependent DNA methylation processes at centromeric repeats, indicating that epigenetic histone methylation and DNA methylation regulate each other in balance (Pal and Tyler, 2016).

#### 3.2.2 Histone acetylation

Histone acetylation, as an important epigenetic modification, is able to influence chromosome remodeling. Histone acetylation and deacetylation are catalyzed by histone acetyltransferase (HATS) and histone deacetylase (HDAC), respectively. In response to HAT, the acetyl group of acetyl-coenzyme A (AcCoA) translocates to specific lysine residues at the N-terminus of histones, resulting in the activation of gene transcription and also providing a binding site for “binding motif proteins” with recognition of this histone modification (e.g., reading protein BRDs). Deacetylation is accomplished in response to HDACs and induces silencing of genes (Fritz, 2013). In the nucleus, the two are in a dynamic balance and precisely regulate gene transcription and expression (Pal and Tyler, 2016).

Cyclic adenosine monophosphate effector-element-binding protein (CREB) binding protein (CBP) and p300 are one of the HATs, and P300/CBP-associated factor (PCAF) induces autophagy by acetylating histone H4 while inhibiting the Akt/mTOR signaling pathway. Because p300 transcription promotes cell cycle progression, chromatin remodeling, and activates DNA repair pathways, inhibition of p300 can arrest cell cycle progression and induce cellular senescence. Levels of CBP acetylated core histones and transcription factors, especially H4K5, decreased with age, whereas CR could increase their levels. PCAF has been shown to promote autophagy and inhibit the growth of hepatocellular carcinoma by inhibiting the Akt/mTOR signaling pathway (Jia et al., 2016). Mammalian cells were starved and rapidly depleted of AcCoA, resulting in decreased activity of acetyltransferase P300, as well as induction of autophagy (Marino et al., 2014).

HDACs are divided into two families: the classical HDAC family (HDACI to HDACII) and the NAD^+^ dependent HDAC family (SIRT1-SIRT7). HDAC is involved in regulatory functions and gene expression in many cells through the interaction of many different transcription factors. Reducing histone acetylation levels in *Drosophila* has been found to significantly prolong its lifespan (Peleg et al., 2016). Compared with young mice, HDAC activity was significantly increased in aged mice, and the use of HDAC inhibitors in aged mice could delay aging. HDAC inhibition can lead to transcriptional activation of several key tumor-related genes such as p21WAF1/CIP1, P53, GATA-1 and α-estrogen receptor, which help to inhibit cancer proliferation and induce differentiation (Victoria et al., 2000). Sirtuins are present in various organisms from yeast to mammals and are involved in the regulatory function of cells and epigenetic regulation of specific genes (Bradshaw, 2021). Evidence suggests that some of the beneficial effects of CR on increasing lifespan and improving health are modulated by mechanisms involving sirtuins, particularly SIRT1 (Lin et al., 2000; Cohen et al., 2004; Nemoto et al., 2004). Li et al. mimicked CR in cells by reducing glucose concentrations in cell growth medium, and experimentally demonstrated that glucose restriction can directly activate SIRT1, which can bind to the p16INK4a gene promoter, resulting in chromatin remodeling of the gene promoter and decreased expression of this gene significantly prolonging cell life. And p16 is a well-known aging-related gene (Li and Tollefsbol, 2011). In addition to sirtuins, other histone-modifying enzymes are also affected by CR. CR increased histone H3 Lys9 acetylation in aged mice (Cao et al., 2018). Mice remained CR from weaning onwards and prevented age-related increases in HDAC2 in the hippocampus, particularly in the CA3 and CA1-2 subregions (). Whereas in *S. cerevisiae*, CR prolonged the lifespan of yeast by significantly reducing N-alpha-terminal acetyltransferase (Nαt4) expression and causing loss of histone H4 N-terminal acetylation (Molina-Serrano et al., 2016) (Table 1). In addition, AMPK plays an important role in histone acetylation. It is able to phosphorylate the H2BS36 site and activate specific HATs, thereby enhancing acetylation of histones. And nuclear AMPK is able to phosphorylate HDAC4 and HDAC5, thereby promoting histone acetylation, while increasing AcCoA and NAD^+^ levels, which act as an important signaling pathway for aging regulation (Mannick et al., 2014). This evidence suggests that deacetylation may be a protective mechanism against nutritional stress and may influence the development of degenerative diseases associated with aging during aging (Dubey et al., 2018).

### 3.3 Non-coding RNA

The ncRNAs include various RNAs such as microRNAs (miRNAs), piRNAs, and long non-coding RNAs (lncRNAs). MiRNAs are a broad class of small non-coding RNAs that are able to play a key role in the post-transcriptional regulation of genes by binding the 3‘untranslated region of mRNAs of specific genes, and may be involved in the process of aging (Inukai et al., 2012; Hooten et al., 2013; Huan et al., 2018). Maes et al. predicted miRNAs related to the aging process in mouse liver and did not find any miRNAs significantly downregulated with age. Interestingly, targets of upregulated miRNAs (miR-669c, miR-709, *etc.*) were associated with detoxification activity and regenerative capacity functions known to decline in aged liver (Maes et al., 2008). CR can affect the expression of age-related miRNAs in different tissues. CR inhibited the increase of a large number of miRNAs in mouse serum (Dhahbi et al., 2013) and also counteracted the increase of miR-181a-1, miR-30e and miR-34a in mouse brain, resulting in increased expression of Bcl-2 and reduced apoptosis (Khanna et al., 2011). Wood et al. also found that miR-98-3p was significantly highly expressed in the cerebral cortex of rats under CR conditions (Wood et al., 2015). In rhesus monkey skeletal muscle, CR was able to suppress age-induced increases in miR-451 and miR-144 levels, rescuing levels of miR-181b and shifting it toward a younger phenotype (Evi et al., 2013) (Table 1). LncRNAs also play an important role in cellular senescence. During aging, stress resistance decreases and lncRNAs are differentially expressed. LncRNAs can regulate the function of mouse hematopoietic stem cells and affect cell self-renewal and differentiation (Sommerkamp et al., 2019). Clearly, changes in ncRNAs levels during aging are also a means of regulating target gene expression, but the relationship between CR and it has been less investigated.

## 4 Interaction between autophagy and epigenetic modifications in caloric restriction

Some studies have shown that there is a relationship between autophagy and epigenetic modifications. Autophagy has multiple physiological and pathological roles in cellular functions, is triggered by oxidative stress, and is regulated by PI3K/mTOR/p70S6 kinase (p70S6K) and extracellular signaling pathways. Arsenite increased the number of autophagosomes and p70S6K, whereas 5-aza-deoxycytidine, a DNMT1 inhibitor, abolished the effect of arsenite on p70S6K expression (Huang et al., 2015). In macrophages from aging mice, promoter regions of autophagy-related genes Atg5 and LC3 were highly methylated. Methyltransferase inhibitor-EGCG treatment restored Atg5 and LC3 expression to trigger autophagy *in vivo* and *in vitro* (Khalil et al., 2016). Histone methyltransferase G9a (*drosophila* histone methyltransferase G9a, dG9a) in *Drosophila* maintains energy levels during starvation, and consumed dG9a inhibits starvation-induced autophagy by controlling Atg8a (An et al., 2017). Hi Jai et al. suggested that activation of specific epigenetic programs is essential for sustained autophagic responses and suggested that AMPK-SKP2-CARM1 histone arginine methyltransferase signaling could serve as a potential therapeutic target in autophagy-related diseases. Nutrient starvation induces protein levels and activity of AMPK in the nucleus. Activated AMPK then phosphorylates FOXO3, leading to SKP2 downregulation and increased levels of CARM1 protein in the nucleus (Shin et al., 2016).

**FIGURE 4:**
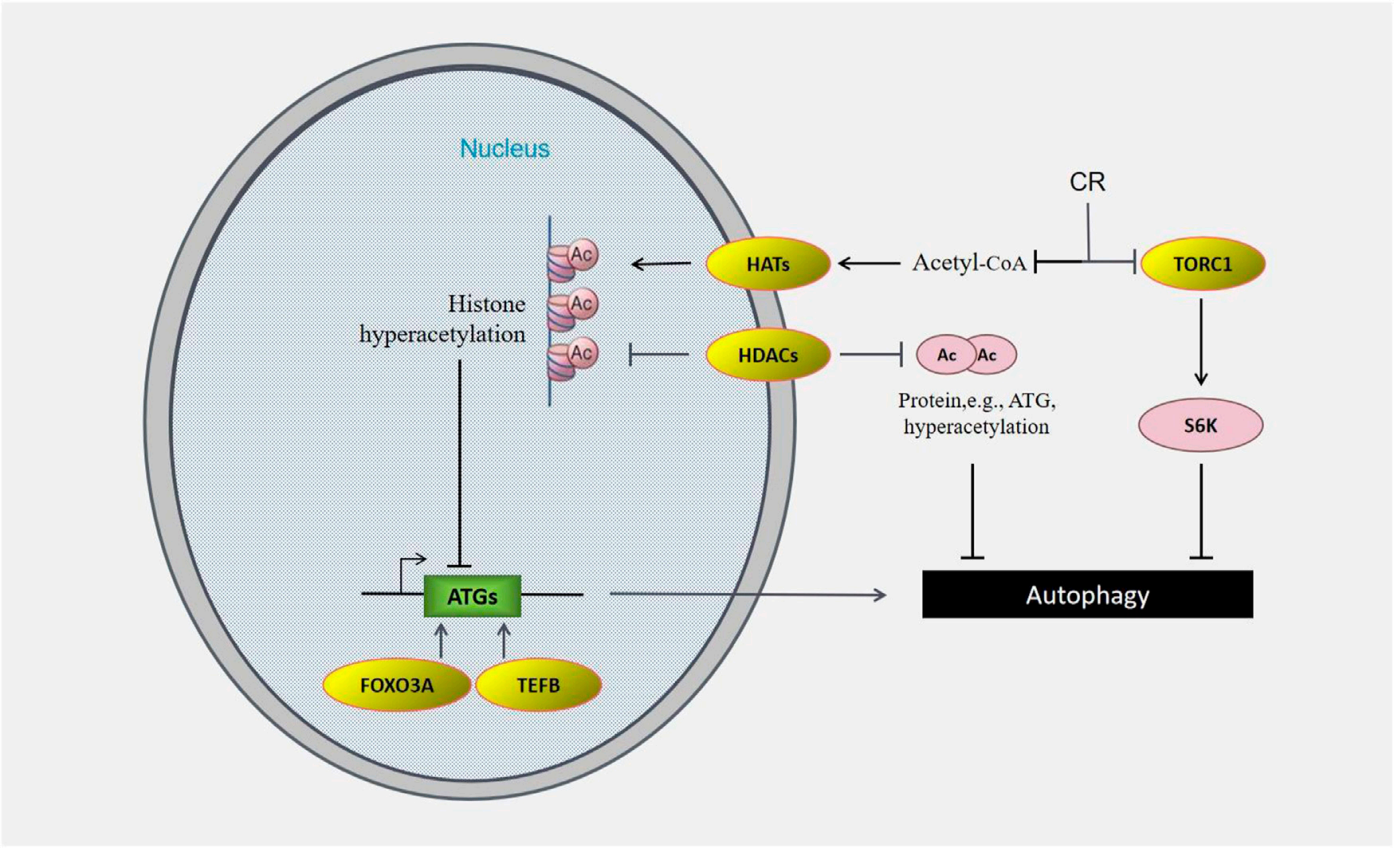
CR regulates epigenetic *versus* autophagic induction. Ac is controlled by levels of HATs such as EP300, HDACs such as SIRT1, and acetyl-CoA. In addition to epigenetic regulation *via* histone acetylation and methylation, transcription factors such as FOXO3a or TFEB also influence transcription of pro-autophagic genes such as ATGs.

Recently, it has been shown that H4K16 acetylation is associated with autophagy activation. When mouse fibroblasts were treated with starvation for autophagy, the degree of H4K16 acetylation was significantly reduced (Fullgrabe et al., 2013). Similar trends were found in human cancer cell lines such as U1810 and HeLa as well as yeasts after the same treatment, while the expression of H4K16 acetyltransferase was also significantly reduced (Fullgrabe et al., 2013). This suggests that the reduced acetylation of H4K16 is significantly associated with the initiation of autophagy in different types of cells. Experiments have demonstrated that PCAF increases acetylation of histone H4 while inhibiting the Akt/mTOR signaling pathway to induce autophagy (Jia et al., 2016). Deacetylation favored autophagy as shown by immunofluorescence detection of acetylated proteins that depletion of AcCoA was accompanied by a reduction in the degree of acetylation of most proteins. CR reduced EP300 activity by depleting AcCoA, which is required for AcCoA mediated inhibition of autophagy (Green et al., 2011). Starvation induced deacetylation of essential autophagy proteins (Atg5, Atg7, Atg12, and LC3) induced by SIRT1 and promoted autophagy (Canto et al., 2009; Yessenkyzy et al., 2020). Magnaporthe oryzae gene GCN5 encodes HAT through acetylation of Atg7 and negatively regulates autophagy caused by light or nitrogen deficiency (Zhang et al., 2017). In addition to epigenetic monitoring, autophagy is controlled by several specific transcription factors (Figure 4). The most important target of SIRT1 on histones is located in H4K16, and it also possesses numerous non-histone sites of action, such as P53 and FOXO3. P53 is one of the human tumor suppressor genes and a suppressor of autophagy. SIRT1 is able to deacetylate lysine 382 at the C-terminus of P53 and reduce the transcriptional activity of P53, thereby regulating autophagy (Brunet et al., 2004). Mammalian members of the FOXO family include FOXO1, FOXO3, FOXO4, and FOXO6, which are involved in regulating life activities such as cell cycle, DNA modification, and autophagy. SIRT1 is able to bind to FOXO3 to form a complex, and under oxidative stress, SIRT1 deacetylates FOXO3 to regulate its activity, while FOXO3 increases the expression of autophagy-related genes LC3 and Bnip3 (Zhao et al., 2010). FOXO1 is required for inducible autophagy in cancer cells, where SIRT2 deacetylates FOXO1 and forms a complex with SIRT2. Under external stimuli or oxidative stress, FOXO1 dissociates from the complex formed by SIRT2 and FOXO1 is reacetylated. Activated FOXO1 specifically binds to Atg7 and activates Atg7, thereby activating autophagic progression (Yi et al., 2012). Thus, SIRTs regulates its transcriptional activity by regulating non-histone acetylation levels, which affects the autophagic process of cells. Aging is also closely linked to circadian rhythms, new research reports. CR retards aging by preserving the biological rhythmic function of stem cells through autophagy and epigenetic modifications (Sato et al., 2017; Solanas et al., 2017). MiRNAs can affect histone modification by targeting and regulating the expression of autophagy-related genes, thereby regulating the occurrence and action of autophagy in cells. For example, miR-30A expression levels are decreased in human cancer cells when nutrients are scarce. And changes in miR-30A activity can cause changes in Beclin one expression levels, which affect autophagic activity (Zhu et al., 2009). MiR101 can regulate the process of autophagy by targeting and degrading autophagy-related genes RAB5A and Atg4D. The target genes degraded by miR101 encode autophagy-related proteins, so miR101 can inhibit the occurrence of autophagy (Varambally et al., 2008; Frankel et al., 2011).

As discussed above, epigenetic inheritance, especially DNA methylation and histone modifications, plays an important role in regulating chromatin structure and expression of key genes, and multiple epigenetic enzyme inhibitors can be seen as autophagic triggers. In recent years, the important effect of histone acetylation on autophagy has led to the realization that the regulation of autophagy is no longer limited to the cytoplasm, and its nuclear regulation is equally important. Although histone acetylation has been shown to regulate autophagy, the effects of other histone modifications on autophagy deserve continued exploration. CR prevents aging and aging-related diseases, in part through autophagy and reversing abnormal aging-related epigenetic alterations. These results provide novel insights into the interplay of epigenetic and CR-induced longevity that may contribute to the proposal of anti-aging approaches. Further understanding of the role of epigenetics in human aging and longevity requires a deeper understanding of the mechanisms of epigenetics in energy-restriction-mediated regulation of longevity.

## 5 CRM: Potential epigenetic therapies for aging-related diseases

CR provides an excellent opportunity to prevent aging-related diseases in humans by affecting epigenetic anti-aging. Natural or synthetic compounds similar to CR, the so-called CRMs: they have molecular, cellular, biochemical, and physiological effects similar to CR, without dietary restriction, and are a class of chemicals with potential life-prolonging effects that act through different pathways. Indeed, there are many validated or potential CRMs that influence epigenetic alterations and induce autophagy (Chung and Chung, 2019). So, CR can be set as a reference point, through its phenotype to help find anti-aging drugs. This part mainly collates these CRMs to extend lifespan or delay the occurrence of aging-related diseases through epigenetic means.

### 5.1 Spermidine

Naturally occurring polyamines are inhibitors of acetyltransferases and also play a beneficial life-prolonging role. Spermidine is a potent acetyltransferase inhibitor that may act by inhibiting protein acetylation in cytoplasmic and nuclear proteins. It is regarded as a CRM. It has been shown that spermidine gradually decreases with increasing age in serum and urine of healthy individuals, and people with high spermidine intake have a lower mortality rate than those on a normal diet (Pegg, 2016; Kiechl et al., 2018). The main mechanism of this effect is spermidine-induced autophagy, and this autophagy is independent of SIRT1 and AMPK1/TOR signaling pathways (Morselli et al., 2011). In budding yeast, spermidine treatment increased levels of autophagy genes Atg7, Atg11, and Atg15 expression (Eugenia Morselli et al., 2009). Spermidine also promotes the expression of the human autophagy transcription factor TFEB, induces autophagy and restores memory B-cell immune responses (Zhang et al., 2019). Induction of autophagy by spermidine is associated with epigenetic inheritance. Spermidine was able to inhibit HAT activity in aging yeast, inhibit oxidative stress and necrosis, and trigger deacetylation of histone H3 (Eisenberg et al., 2009). In addition to its role in general cellular homeostasis, spermidine has been implicated in a variety of health-promoting effects. For example, spermidine feeding can prolong the life span of mice and improve stem cell function in the muscle of aged mice; dietary spermidine supplementation can enhance human immunity, prevent bone and joint inflammation, and protect the heart and nerves, which has received much attention from aging researchers (Sacitharan et al., 2019; Karacay et al., 2022). Triethylenetetramine (also known as TETA) is a polyamine synthesized structurally similar to spermidine, which has no effect on the lifespan of mice, but does induce autophagy and reduce weight gain, glucose intolerance, and osteopathy in mice fed a high-fat diet (Castoldi et al., 2020). Experiments have shown that TETA leads to depletion of intracellular AcCoA, deacetylation of cytoplasmic proteins, and activation of autophagy through a novel mode of action, namely through activation of spermidine/spermine N1-acetyltransferase 1 (SAT1) (Pietrocola et al., 2020). In conclusion, TETA may serve as a caloric restriction mimetic with novel modes of action.

### 5.2 Polyphenols

Polyphenols are another class of chemicals that have received attention because of their anti-aging ability. These compounds are found in plant foods, and they exhibit a wide range of biological activities, including antioxidant, anti-inflammatory, anticancer, and anti-atherosclerotic activities. Numerous studies have shown that some plant polyphenols can modulate several mechanisms of CR prolonging effects and are capable of causing epigenetic changes.

Resveratrol is one of the most studied CRMs. In 2004, Wood et al. reported that resveratrol can prolong the lifespan of yeast, nematodes, and flies by activating acetylases, stimulating an upsurge in resveratrol anti-aging research (Jason et al., 2004). Resveratrol not only induces SIRT1 activation to modulate insulin signaling, but also extends lifespan and prevents age-related diseases in part by regulating NF-κB signaling and MAPK (Bhullar and Hubbard, 2015). Matrix metalloproteinase 9 (MMP9), an endoprotease involved in inflammation-induced tissue remodeling, is hyperactivated in CVD, neurodegeneration, and diabetes. Resveratrol activates SIRT1 and downregulates MMP9 expression (Gao and Ye, 2008). In studies on tumor cell lines, resveratrol was observed to inhibit the NF-κB inflammatory response pathway and to reduce miR-221 expression (Wu and Cui, 2017). Although resveratrol had no apparent effect on the mean or median lifespan of mice, it produced a wide range of health benefits for cardiovascular disease, diabetes, and neurological disease (Berman et al., 2017). However, there are relatively few clinical studies on resveratrol in China, and its effectiveness in clinical trials cannot be predicted at present. Resveratrol, there is also a need to explore the potential preventive activity and duration and dose of uptake of resveratrol in clinical trials. It has been shown that curcumin combined with resveratrol can reduce muscle and bone mass damage caused by highly oxidative damage (Murillo Ortiz et al., 2019).

Curcumin, as a CRM, has been found to have various biological activities such as antioxidant, anti-inflammatory, anti-rheumatic, anti-cancer, and anti-neurodegenerative activities, and can treat a variety of chronic diseases. Some studies have shown that curcumin is metabolically protective by regulating DNA methylation and histone acetylation. For example, In a rat model of non-alcoholic fatty liver disease (NAFLD), curcumin treatment significantly reversed DNA methylation levels, increased expression of peroxisome proliferator activated receptor alpha (PPAR -α) and protein, and improved lipid accumulation in the model (Li et al., 2018b). Curcumin is effective in the treatment of myocardial infarction by inhibiting p300-HAT activity and downstream GATA4, NF-κB, and TGF-β-Smad signaling pathways, resulting in decreased expression of cardiac hypertrophy genes (Li et al., 2008; Morimoto et al., 2010). In addition to HAT inhibition, curcumin also improved vascular structure by inhibiting the expression of HDAC1, thereby promoting tissue inhibitor of metalloproteinase-1 TIMP1 transcriptional activation and inhibiting the expression of MMP-2 and TGFβ (Hu et al., 2017). These data suggest that curcumin is a class of chemicals with potential life-prolonging and aging-related disease prevention effects. Although a large number of experiments have shown that curcumin has anti-aging effects and also has the effect of treating aging-related diseases, clinical experimental data on curcumin are still very limited, and data and validation of long-term responses are lacking. In addition, curcumin has low solubility, rapid metabolism, and is difficult to reach effective concentrations in the human body, which is also an urgent problem to be solved.

In addition to the above two compounds, quercetin is also one of the well-studied natural polyphenols. Quercetin can inhibit oxidative damage induced by oxidized LDLoxldl in endothelial cells by activating SIRT1 and regulating AMPK signaling (Hung et al., 2015), and can also regulate the expression of various chromatin modifiers in a dose-dependent manner, such as reducing the activity of DNMT and HDACs and down-regulating global DNA methylation levels (Kedhari Sundaram et al., 2019). Fisetin has anti-inflammatory, neuroprotective, anticancer, and antidiabetic effects, and its regulatory effects on oxidative stress during aging are unknown. In rats, oral administration of phenastine for 6 weeks suppressed aging-induced increases in reactive oxygen species, erythrocyte apoptosis, lipid peroxidation, and protein oxidation levels (Naeimi and Alizadeh, 2017; Singh et al., 2019). Therefore, a diet rich in fesoterone may be a potential anti-aging intervention strategy. 4,4′-Dimethoxychalcone (DMC), although it extends the lifespan of yeast, nematodes, and flies, DMC induces autophagy, independent of mTOR, but rather related to GATA transcription factors (Carmona-Gutierrez et al., 2019). DMC may therefore act synergistically with other CR mimetics against mTOR. Anthocyanin-rich blueberry extract can reduce HAT activity, reduce TNFα signaling and pro-inflammatory gene expression in the liver, regulate gene expression, block liver fibrosis, and protect liver function in aging mice by increasing histone H3 acetylation at lysine residues on histones K9, K14, and K18 (Wei et al., 2017; Zhan et al., 2017). Anthocyanins therefore modulate HDAC and HAT activity to suppress inflammation-related diseases.

### 5.3 Rapamycin

Rapamycin is a major inhibitor of the mTOR signaling pathway. Rapamycin not only increases life span, but also improves quality of life and health. Studies have shown that rapamycin treatment can prolong the life span of invertebrates and mice, slow down or even reverse a variety of age-related changes in mice including arterial function changes, cardiomegaly, periodontitis, immunosenescence, and cognitive decline (Li et al., 2014; Laberge et al., 2015). Interestingly, Anisimov et al. reported a greater increase in mean lifespan in females treated with rapamycin (Anisimov et al., 2011). These results suggest that prolonging human healthy lifespan by rapamycin is a feasible strategy, however, unfortunately, rapamycin is also able to inhibit TORC2 in multiple tissues including liver, adipose tissue, and skeletal muscle (Lamming LY et al., 2012). So long-term use of rapamycin produces many side effects, including increased risk of cataracts, viral and fungal infections, and joint pain, which limit its use to delay aging in healthy individuals. In addition, the dosage is also a problem, and humans usually take rapamycin at doses of 2–5 mg per day, while laboratory mice take doses as high as 2.24 mg/kg per day. Thus, the use of safer, but possibly weaker, indirect mTORC1 inhibitors, such as metformin and resveratrol, may prove useful. However, some researchers believe that rapamycin may not be a true CRM, and the combination of dietary restriction and rapamycin may work better than either alone (Strong et al., 2016).

### 5.4 Metformin

Metformin is a biguanide antidiabetic agent. It has been shown that metformin, in addition to its proven efficacy as a hypoglycemic agent, can exert multiple anti-aging effects at the cellular and tissue levels is also a traditional CRM (Barzilai et al., 2016). Studies have shown that people with type 2 diabetes taking metformin have longer survival than people who are not diabetic and do not take the drug (Bannister et al., 2014). Metformin also reduces the incidence of cardiovascular disease, increases cognitive function, and improves age-related dysfunction (Solymar et al., 2018). The mechanisms by which metformin may exert its anti-aging effects have been investigated (Wyss-Coray, 2016): inhibition of mitochondrial electron respiratory chain complex 1, reduction of ROS production and inhibition of JNK signaling (McHugh and Gil, 2018); activation of AMPK signaling pathway, inhibition of mTOR (Fontana et al., 2018); inhibition of NF-κB to reduce inflammatory responses; and (Ogrodnik et al., 2017) reduction of insulin levels and IGF-1 signaling, reduction of glucose absorption in the gastrointestinal tract and increased insulin sensitivity (Batandier et al., 2006; Nair et al., 2014). Metformin favorably impacts metabolic and cellular processes, all of which impact cell proliferation, senescence, stress defense, autophagy, protein synthesis, and inflammation that are strongly age-related. Metformin can also affect epigenome and gene expression to influence aging. For example, metformin induced HAT1 phosphorylation and increased its activity in a mouse embryonic fibroblast model (Marin et al., 2017). HDACs can deacetylate histones, various transcription factors, and regulatory proteins, thereby directly or indirectly affecting glucose metabolism. Following metformin treatment, HDAC4, 5, and 7 activity in rat liver was inhibited, followed by an increase in global H3 acetylation levels (Khan and Jena, 2016). In human umbilical vascular endothelial cells, metformin treatment increased the NAD^+^/NADH ratio, thereby increasing SIRT1 gene expression and reducing signs of cellular senescence and reactive oxygen species production (Zhang et al., 2015). Similarly, metformin can improve ovarian reserve and delay the aging process in mice by inducing SIRT1 expression and reducing oxidative damage (Xian Qin et al., 2019). However, metformin may also produce undesirable side effects, and exposure to metformin has been reported to be time- and dose-dependent, with chronic use resulting in adverse events such as increased cognitive impairment and vitamin B12 deficiency in patients with type 2 diabetes and increased lactate levels in mice and humans (de Jager et al., 2010; Madiraju et al., 2014; Kuan et al., 2017). In general, metformin decreased the activity of most HATS, HDACs, and DNMT and increased the activity of HAT1 and SIRT1 through AMPK-mediated phosphorylation. But it is difficult to generalize the effect of metformin on epigenome and gene expression.

### 5.5 Aspirin

Aspirin, a non-steroidal anti-inflammatory drug, is also considered a mimetic of CR. Aspirin has been shown to extend median lifespan in nematodes, flies, and mice. In *C. elegans*, aspirin increases lifespan by activating AMPK and stimulating the Dad-16/FOXO3 signaling pathway (Wan et al., 2013). The effect of aspirin on the epigenome may also serve as a potential mechanism. Data suggests that aspirin can reverse tumor suppressor gene hypermethylation in cancer tissue, affect deacetylase activity, and may also downregulate miRNAs with oncogenic-like function or upregulate miRNAs with tumor suppressor-like function. In particular, DNA methylation alterations, are considered to be one of the most common molecular alterations in human tumors. Studies have shown that aspirin can inhibit DNA methylation and epigenetic aging in healthy colon over time, thereby reducing the risk of cancer (Noreen et al., 2014). In contrast, in the ASPREE (a randomized double-blind placebo-controlled trial) trial, daily low-dose aspirin in older adults without known disease did not reduce cardiovascular disease-related mortality and increased bleeding and all-cause mortality (McNeil et al., 2018a; McNeil et al., 2018b). In addition, long-term use of aspirin may cause side effects such as stomach bleeding and brain bleeding, and the older the age, the greater the possibility of internal bleeding, and it is recommended that the doctor should agree before taking it.

## 6 Other dietary interventions

### 6.1 Intermittent fasting (IF)

Among the available dietary anti-aging interventions, in addition to CR, different forms of IF have gained scientific and public interest because of their broad health-promoting properties. IF includes different rhythmic or recurrent rhythmic fasting regimens, such as alternate-day fasting, periodic fasting, and time-limited eating. Although there are fewer reports on IF than CR, recent studies clearly show that IF also extends the lifespan of vertebrate and invertebrate model organisms (Honjoh et al., 2009; Fontana and Partridge, 2015; Catterson et al., 2018; Nencioni et al., 2018; de Cabo and Mattson, 2019). In fact, CR and IF also lead to common metabolic and physiological changes in multiple tissues and organs (Francesco et al., 2018). For example, ketone bodies, increased insulin sensitivity, and decreased IGF-1. A study showed that caloric restriction and intermittent fasting both reduced body weight and glycosylated hemoglobin levels in obese diabetic patients at risk for cardiovascular disease. Both regimens reduce overall inflammatory response and oxidative stress and also elicit similar behavioral changes, such as increases in hunger response and cognitive response (Francesco et al., 2018). Therefore, it is generally accepted that common molecular mechanisms may mediate the process of lifespan extension by CR and IF. However, there are also independent mechanisms between CR and IF. A major difference is that IF may extend lifespan without reducing calorie intake overall by exploiting molecular pathways in response to fasting (Anson et al., 2005). However, a comprehensive understanding of the mechanisms by which IF extends lifespan remains lacking. Because it is difficult to control parameters such as size and time/duration of food intake in lower organisms, such as yeast, worms, and flies, these animals are basically fed *ad libitum* when food is obtained regardless of the method chosen, and their food consumption is not easily measured. So far, it has not been possible to determine whether the effects observed with CR alone and fasting can be seen as completely independent of each other. At the same time, whether IF may have a better effect than CR has been highly debated.

### 6.2 Methionine restriction (MR)

CR reduces oxidative stress and increases lifespan in many species. Limiting essential amino acid intake is a critical medium for the life-prolonging effects of CR, including limiting methionine intake (Gallinetti et al., 2013). Methionine is an essential amino acid for normal growth and development of the human body and is involved in protein synthesis, functional regulation, and DNA methylation (Levine et al., 1996; Luo and Levine, 2009). In humans, methionine can be obtained from food or gastrointestinal microorganisms. MR has been shown to prolong the healthy lifespan of different model organisms (Richie et al., 1994; Miller et al., 2005; Cabreiro et al., 2013; Lee et al., 2014) and has beneficial effects such as reducing cancer, increasing insulin sensitivity, and reducing inflammation and oxidative stress (Wanders et al., 2014; Liu et al., 2017; Wanders et al., 2017; Yang et al., 2019; Wanders et al., 2020). The life-extension effect of MR has been attributed to many different mechanisms.

A critical component of the beneficial effects of MR on cell survival and longevity is activation of autophagy. The autophagic process depends on the proteolytic activity of vacuoles, which is determined by vacuolar acidification (Nakamura et al., 1997). Ruckenstuhl et al. found that MR prolonged yeast lifespan in an autophagy-dependent manner. Single deletion of several core autophagy factors (Atg5, Atg7, or Atg8) could completely abolish the longevity ability of MR. MR enhances vacuum acidity, a phenotype that essentially requires autophagy (Ruckenstuhl et al., 2014). Sutter et al. also showed that methionine regulates mTORC1 signaling pathway and autophagy by regulating the methylation status of phosphatase 2A (PP2A) in yeast (Sutter Benjamin et al., 2013; Laxman et al., 2014). SIRT1 can also affect autophagy. The effect of MR on SIRT1 may be related to age. MR (.172%) showed increased expression of the SIRT1 gene in 12-month-old mice, but not in 2-month-old mice (Lees et al., 2014). MR (.516%) was performed in 24-month-old Wistar rats, which showed reduced SIRT1 expression at the protein level (Sanchez-Roman et al., 2012). Fibroblast growth factor-21 (FGF-21) is one of the most important growth factors and plays an important role in the pathogenesis of metabolic diseases such as obesity and diabetes (Xu et al., 2009). In obese mice, MR (.12%) increased FGF-21 levels and was more resistant to glucose and insulin (Ables et al., 2012). SIRT1 activates PPARα and PGC-1α in mice and then stimulates FGF-21 synthesis and fatty acid oxidation (Purushotham et al., 2009). But the increase of FGF-21 was not associated with SIRT1 expression in Lee et al. (Lees et al., 2014). Thus, MR-induced synthesis of FGF-21 is only some of them SIRT1-dependent. These imply the potential value of dietary MR in preventing diabetes. In addition, FGF-21 also inhibited the IGF-1 pathway in mice, but had no effect on hepatic IGF-1 production in 2-month-old mice (Lees et al., 2014).

MR can reduce cancer morbidity and mortality. One study showed that among all rats transplanted with Walker‘s tumors, tumor growth was significantly reduced in rats fed diets containing no methionine, valine, or isoleucine (Sugimura et al., 1959). In a subsequent study published in 1974, cancer cells and normal cells were cultured in culture medium, and the growth of malignant cells was significantly impaired in methionine-free culture medium, while the growth of normal cells did not change. Thus, MR has the ability to alter cancer cells while maintaining normal, healthy cells (Cavuoto and Fenech, 2012). Similarly, MR suppressed prostate cancer development in mouse models of prostate cancer, particularly in the anterior and dorsal lobes where prostate cancer was most severe (Sinha et al., 2014). In a nude mouse model inoculated with MCF10AT1 breast cancer cells, MR0.12% inhibited tumor progression in mice by reducing cell proliferation and increasing apoptosis (Hens et al., 2016).

Recently, the effects of nutrients on gene expression have aroused high concern among scientists around the world. Epigenetic alterations have been suggested as a potential mechanism for metabolic phenotype switching in methionine restriction. However, the effect of DNA and histone modifications on the beneficial effects of methionine restriction is unknown. Studies in adult mice have shown that MR (.12%) lasting 12 weeks leads to increased hepatic DNA methylation, but has opposite effects on adipose tissue (Mattocks et al., 2017). Methionine restriction can also alter histone methylation and acetylation in humans (Mentch et al., 2015). Several miRNAs are also involved in nutrient sensing and regulatory signaling pathways, and they also change when performing MR. For example, limiting methionine (.12%) increased miR-31 levels in mouse plasma and liver (Plummer et al., 2017; Latimer et al., 2018). However, there is no clear evidence on the role of miRNAs in the life-prolonging effects of methionine-restricted diets.

## 7 Conclusion

Prolonging health or longevity is beneficial at both the individual and social levels. However, so far, only a few experimental settings have been shown to extend mammalian lifespan. Autophagy and epigenetic modification are multigene regulatory processes that play an important role in regulating aging as well as aging-related diseases. Autophagy, such as a double-edged sword, can maintain cell survival and delay aging, but excessive autophagy can lead to cell death and promote aging. Therefore, how to precisely regulate and activate autophagy while clarifying the interaction between autophagy and epigenetic modifications may contribute to the proposal of anti-aging methods. Epigenetic modification, unlike DNA mutations, is a reversible regulation. CR prevents aging and aging-related diseases, in part through autophagy and reversing abnormal aging-related epigenetic alterations. This suggests that epigenetic modification is expected to be a potential therapeutic strategy against aging and its aging-related diseases. Further understanding of the role of epigenetics in human aging and longevity requires a deeper understanding of the influence of the external environment on epigenetics, such as the mechanism of epigenetics in CR-mediated longevity regulation.

In recent years, although autophagy and epigenetic modifications induced by CR have been experimentally confirmed in delaying aging and preventing some age-related degenerative diseases, the degree, time limit, food composition and study subjects of CR are different, which may have an impact on its biological effects. In humans, the effects of nutritional interventions are related to factors such as specific populations, modes of intervention, and genetics. We also need a deeper understanding of epigenetic mechanisms in the regulation of energy restriction-mediated longevity. In preclinical studies, fasting and CR have been shown to prolong life and health, induce autophagy, and improve symptoms of various diseases. However, the transition to the clinic has been slow, and hunger, compliance, and health during fasting are also issues to consider. Importantly, long-term and follow-up trials are needed to assess their long-term efficacy and safety. In mice, for example, chronic CR increases susceptibility to infection, particularly of viral nature. In contrast, restriction of a specific amino acid, namely methionine, has also been shown to greatly enhance autophagy and prolong mammalian lifespan, with the advantage of having good applicability. For practical applications, it may not be so important which dietary intervention produces a better effect, but what dietary intervention the patient is personally suitable and willing to follow.

There are now some clinical data demonstrating that CR prolongs life expectancy in individuals by reducing the risk of aging-related diseases, but may adversely affect certain groups, and optimal intake and duration of long-term CR, receptor sensitivity, and degree of activation of post-receptor pathways remain to be determined in further studies. CR may have a much smaller benefit on human lifespan than animals and may be more useful than prolonging lifespan in reducing functional decline and preventing chronic diseases. In addition, because the study of long-term high-intensity CR in humans is very limited, CR drugs and compounds that can replace CR have also received attention in recent years, such as metformin and rapamycin, but they will also produce inevitable toxic side effects. Identifying new, secure CRMs will therefore be important. At present, CRM is only a drug used alone, and whether multiple CRMs can be used in combination, or interact with other anti-aging interventions (such as exercise) to exert more prominent therapeutic effects remains to be explored.
